# Physical Performance Measures in Older Women with Urinary Incontinence: Pelvic Floor Disorder or Geriatric Syndrome?

**DOI:** 10.1007/s00192-020-04603-y

**Published:** 2020-11-17

**Authors:** Tatiana V.D. Sanses, Sharee Pearson, Derik Davis, Chi Chiung Grace Chen, Soren Bentzen, Jack Guralnik, Holly E. Richter, Alice S. Ryan

**Affiliations:** 1 Department of Obstetrics and Gynecology, Howard University College of Medicine;; 2 Department of Diagnostic Radiology and Nuclear Medicine, University of Maryland School of Medicine;; 3 Department of Gynecology and Obstetrics, John Hopkins University School of Medicine;; 4 Department of Epidemiology and Public Health, University of Maryland School of Medicine;; 5 Department of Obstetrics and Gynecology, University of Alabama School of Medicine;; 6 Department of Medicine, University of Maryland School of Medicine

**Keywords:** Urinary Incontinence, Mobility, Physical Performance Measures, Geriatric Syndrome, Functional Impairment

## Abstract

**Objective:**

To evaluate physical performance measures of mobility and functional impairments and assess their association with urinary incontinence (UI) severity and impact on quality of life among older women with UI.

**Methods:**

In a cross-sectional pilot study, 20 women aged ≥ 70 years with UI completed UI questionnaires (Global Impression of Severity, Incontinence Impact Questionnaire (IIQ-7)) and functional status evaluation. Functional status evaluation included the Modified Physical Performance Test (MPPT; range 0–36), Short Physical Performance Battery (SPPB; range 0–12), and other physical performance measures (e.g., Timed Up and Go [TUG]). MPPT and SPPB scores <32 and <10, respectively, indicated impaired mobility and function. Descriptive statistics and spearman correlation coefficients evaluated study variables and associations between UI and physical performance measures.

**Results:**

Women were 76.6±4.7 years old with mean body mass index 33.5±9.0 kg/m^2^. Mixed UI was the most prevalent (n=17; 85%), and 14 (70%) participants rated their UI as moderate or severe. Low MPPT (<32) and SPPB (<10) scores were present in 65% (n=13) and 35% (n=7) of participants, respectively. Lower MPPT score (r=−0.46; P=0.04) and worse TUG performance (r=0.50; P=0.03) were associated with greater UI impact on quality of life based on IIQ-7. SPPB did not correlate (P>0.05) with UI measures.

**Conclusions:**

Mobility and functional impairments are common among older women with UI. Associations between MPPT score, TUG performance with UI impact on quality of life suggest these physical performance measures could be markers of mobility and functional impairments in future research on UI in older women.

## Brief Summary

Further research in utility of physical performance measures as markers of mobility and functional impairments in older women with urinary incontinence is warranted.

## Introduction

Urinary incontinence (UI) is the most common pelvic floor disorder present in more than 30% of women aged 80 years and older [1]. In addition, the prevalence of UI geriatric syndrome among women aged 65 years and older in Women’s Health Initiative Study was 29.3% [2]. While significant progress has been made in understanding UI, International Urogynecological Association (IUGA)/International Continence Society (ICS) joint report on the terminology for female pelvic floor dysfunction does not define UI pelvic floor disorder versus geriatric syndrome among older women [3].

Geriatric syndrome is defined as a “multifactorial health condition that occurs when the accumulated effect of impairments in multiple systems render an [older] person vulnerable to situational challenges”[4, 5] emphasizing “multiple causation of a unified manifestation”[6, 7]. Therefore, UI geriatric syndrome in women could be considered when accumulated impairments in multiple systems compromise [an older] woman compensatory ability to control urine regardless of whether any specific urologic pathology exists. Recent research suggests four shared risk factors across most common geriatric syndromes, age ≥ 65 years, impaired mobility, functional and cognitive impairments [5]. DuBeau called for an alteration of the current treatment paradigm for UI to a stepped-care approach based on different age groups emphasizing that UI in older persons may be caused by factors other than only lower urinary tract abnormalities [8]. However, contemporary clinical approach to treat UI in older women typically does not include evaluation for these factors, and hence miss salient non-urologic factors that may be contributing to UI in older women.

While impaired mobility and functional impairments are risk factors for UI geriatric syndrome, current literature is not consistent on the best marker of mobility and functional impairments in older women with UI [9–13], thus limiting our ability to understand UI geriatric syndrome. Physical performance measures are used to better understand the health of older people, assess “building blocks” of functioning, and are powerful predictors of adverse outcomes including disability, nursing home admissions, and mortality [14–16]. Physical performance measures may be an ideal component of a broader UI clinical assessment to examine mobility and functional impairments in older women with UI. We hypothesized that older women with more severe UI symptoms and impact on QOL would have worse physical performance , and some physical performance measures would have stronger associations with UI severity than others. Therefore, the study objectives were 1) to assess physical performance measures among older women with UI and 2) to evaluate their association with UI severity and impact on quality of life (QOL) for possible markers of mobility and functional impairments in future clinical research in older women with UI.

## Materials and Methods

### Study Design and Sample

This cross-sectional pilot study was conducted at the University of Maryland Claude D. Pepper Older Americans Independence Center. Institutional Review Boards at the University of Maryland Baltimore (#00066737) and Johns Hopkins Bayview Medical Center (#00108819) approved the study protocol, and all participants provided written informed consent. Women age 70 and older with symptomatic UI were invited to participate through advertisement and Urogynecology clinics at University of Maryland Medical Systems, Baltimore VA Medical Center, and Johns Hopkins Bayview Medical Center. Participants were recruited prospectively from 5/2016 through 7/2017 after IRB approval.

Women who expressed initial interest in the study from either clinics or advertisements were screened by research staff and then invited for an in-person visit and consent. Participants completed urogynecologic evaluation and 3-day bladder diary. Inclusion criteria were 1) age ≥ 70 years, 2) symptomatic UI ≥ 3 months, and 3) UI confirmed on a 3-day bladder diary. Exclusion criteria included 1) inability to follow up or complete a bladder diary, 2) cognitive impairment (mini-mental state examination score <25) [17], 3) post-void residual ≥ 150 ml, 4) non-ambulatory (wheelchair bound), unable to complete physical performance measures due to any uncontrolled medical condition (e.g., congestive heart failure, uncontrolled diabetes, etc.), neurological, and musculoskeletal conditions (stroke, multiple sclerosis, amyotrophic lateral sclerosis, rheumatoid arthritis), 5) UI associated with hematuria, urinary tract infection, fistula, pelvic organ prolapse > stage 2, and fecal impaction. Once study inclusion criteria were met, women underwent physical performance measures and functional status evaluation.

### Urogynecologic evaluation

Urogynecologic evaluation included prior and current UI treatments, and physical examination (pelvic organ prolapse quantification [POP-Q] evaluation, urethral hypermobility (defined as ≥ 30°), vaginal atrophy (present/absent based on presence of pale color of vaginal mucosa and diminished or absent vaginal rugae), cough stress test, urine dipstick, post void residual). UI was classified according to International Urogynecological Association (IUGA)/International Continence Society (ICS) joint report on the terminology for female pelvic floor dysfunction [3]. Symptomatic UI was defined by the presence of UI on bladder diary and responses on validated questionnaires (6-item Urogenital Distress Inventory Short Form [UDI-6], 7-item Incontinence Impact Questionnaire-Short Form [IIQ-7], and Patient Global Impression of Severity [PGI-S]) [18, 19]. The UDI-6 and IIQ-7 yield summary scores on a scale ranging from 0 to 100, where higher scores indicate more severe UI symptoms and greater symptom-specific quality of life impact, respectively. The PGI-S is a 1-item scale to measure UI severity that range from 1 (normal) to 4 (severe). Participants also underwent the two-part Medical Epidemiologic Social Aspect of Aging (MESA) evaluation to characterize stress and urgency UI with scores ranging from 0 to 100 where higher scores indicated more severe symptoms of stress or urgency UI [20]. The type of UI (stress, urgency, or mixed) was determined based on bladder diary and MESA responses.

### Physical Performance Measures and Functional Status Evaluation

Comprehensive evaluation of physical performance measures and functional status was performed by research staff experienced in administering these measures. Women could use any walking aid they used daily, and adequate rest was provided between tests. Women could utilize any walking aid they used daily, with adequate rest provided in between each test, and all assessments were performed with equipment that was standardized between different participants. The evaluation lasted about 1–2 hours. Our previous research showed that highest prevalence of upper, lower body mobility, and strength impairments was in women age ≥ 70 years [21]. Therefore, we utilized physical performance measures to evaluate upper, lower body mobility, and strength. The study protocol included Modified Physical Performance Test (MPPT) [22, 23], Short Physical Performance Battery (SPPB) [15, 16], 6-minute walk [24], Four Square Step Test (FSST) [25], “Timed Up and Go” (TUG) [26], and grip strength [27, 28].

The MPPT included the static balance, chair rise tasks, gait speed over a 50-foot walk, timed ascent of one flight of stairs, ability to ascend and descend four flights of stairs, putting on and removing a jacket, picking up a penny, placing a book on a shelf, and safely turning around 360 degrees [22]. The MPPT component measure scores ranged from 0 to 4 resulting in a maximum possible composite score of 36. The SPPB consisted of three tests: static balance, chair rise, and gait speed, with each assessment rated from 0 to 4, yielding a potential maximum composite score of 12 [15]. Lower MPPT and SPPB scores corresponded to greater functional impairments. Specifically, MPPT and SPPB scores of <32 and <10 indicated impaired mobility and function, respectively [15, 23].

Other physical performance measures to evaluate lower body mobility were the 6-minute walk [24], FSST [25], and TUG [26]. The FSST also evaluated dynamic standing balance [25]. The protocol also included grip strength to measure upper body muscle strength [27]. Physical performance measures protocol is available in Appendix A. Lastly, in addition to physical performance assessments, participants completed functional status evaluation on self-reported limitations in activities of daily living (ADL) and instrumental activities of daily living (IADL) [29].

### Statistical Analysis

Descriptive statistics (means, standard deviations [SD], medians, interquartile ranges [IQR], frequencies, and percentages) were performed to assess demographics, urogynecologic clinical characteristics, functional status and physical performance measures. Means, SD, medians, and IQR were used to evaluate continuous measures, and frequencies and percentages were used to assess categorical variables. Spearman correlation coefficients were used to examine associations between UI and physical performance measures. Correlation coefficients were classified as weak (r <0.3), moderate (0.3 < r > 0.6), or strong (r >0.6). Alpha was set at the 0.05 level, and all statistical analyses were conducted using SPSS (Version 25.0. Armonk, NY: IBM Corp). The sample of 20 participants was planned a priori.

## Results

**Figure 1:**
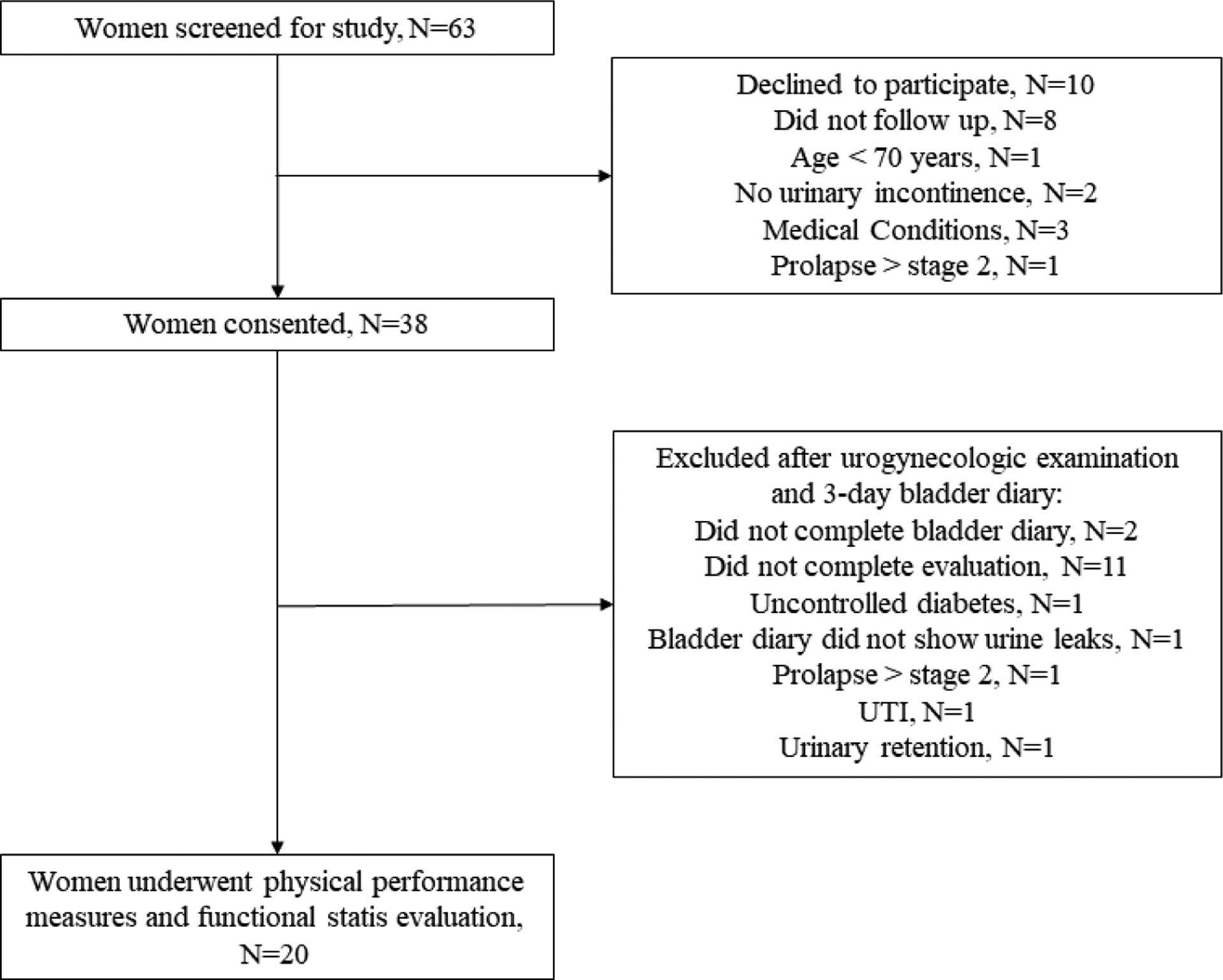
Study flow

A total of 63 women were screened (Figure 1). Out of thirty-eight women consented to participate, 20 participants completed study evaluation. The mean age and body mass index of the study sample (50% white; 90% non-Hispanic) were 76.6 years and 33.5 kg/m^2^, respectively, suggesting participants were older and overweight or obese (Table 1). There was no difference in age among women who were screened but did not complete the study. Most women had prior vaginal deliveries (75%), arthritis (75%), and hypertension (70%), but the prevalence of other comorbid conditions was lower. Participants did not have previous prolapse surgeries. UI symptoms were present for longer than 1 year in 17 (85%) participants, which included 9 (45%) women with symptoms lasting for over 5 years. Despite long-standing UI symptoms, only 7 (35%) women had received any UI treatment. All 7 women received anticholinergic medications for UI treatment, and 1 participant had an anti-incontinence surgery.

Participants had an average of 15.9 UI episodes on the 3-day bladder, and 90% of women self-reported ≥ 2 UI episodes per day (Table 2). Mixed UI was the most common (85%) type of incontinence. By contrast, urgency UI was only present in 15% of women, and no participants had isolated stress UI. Urogynecologic evaluation revealed vaginal atrophy and no urethral mobility in 95% of participants.

Validated UI questionnaires revealed moderate UI severity and impact on QOL (Table 2). PGI-S responses indicated that 12 (60%) and 2 (10%) participants considered their UI as moderate and severe, respectively. UDI-6 demonstrated that 75% of women rated their urinary symptoms as moderate or severe at least in one of the domains. Similarly, IIQ-7 results implied that accidental urine loss moderately affected QOL among 70% of participants at least in one of the questionnaire domains.

The mean MPPT and SPPB scores (Table 2) were 26.6 and 9.4, respectively, and there was a significant positive correlation between these two physical performance measures (r=0.87; P<0.001). Low MPPT (<32) and SPPB (<10) scores were present in 65% (n=13) and 35% (n=7) of participants, respectively. Moreover, every participant with a SPPB score <10 also had a MPPT score <32.

**Figure 2:**
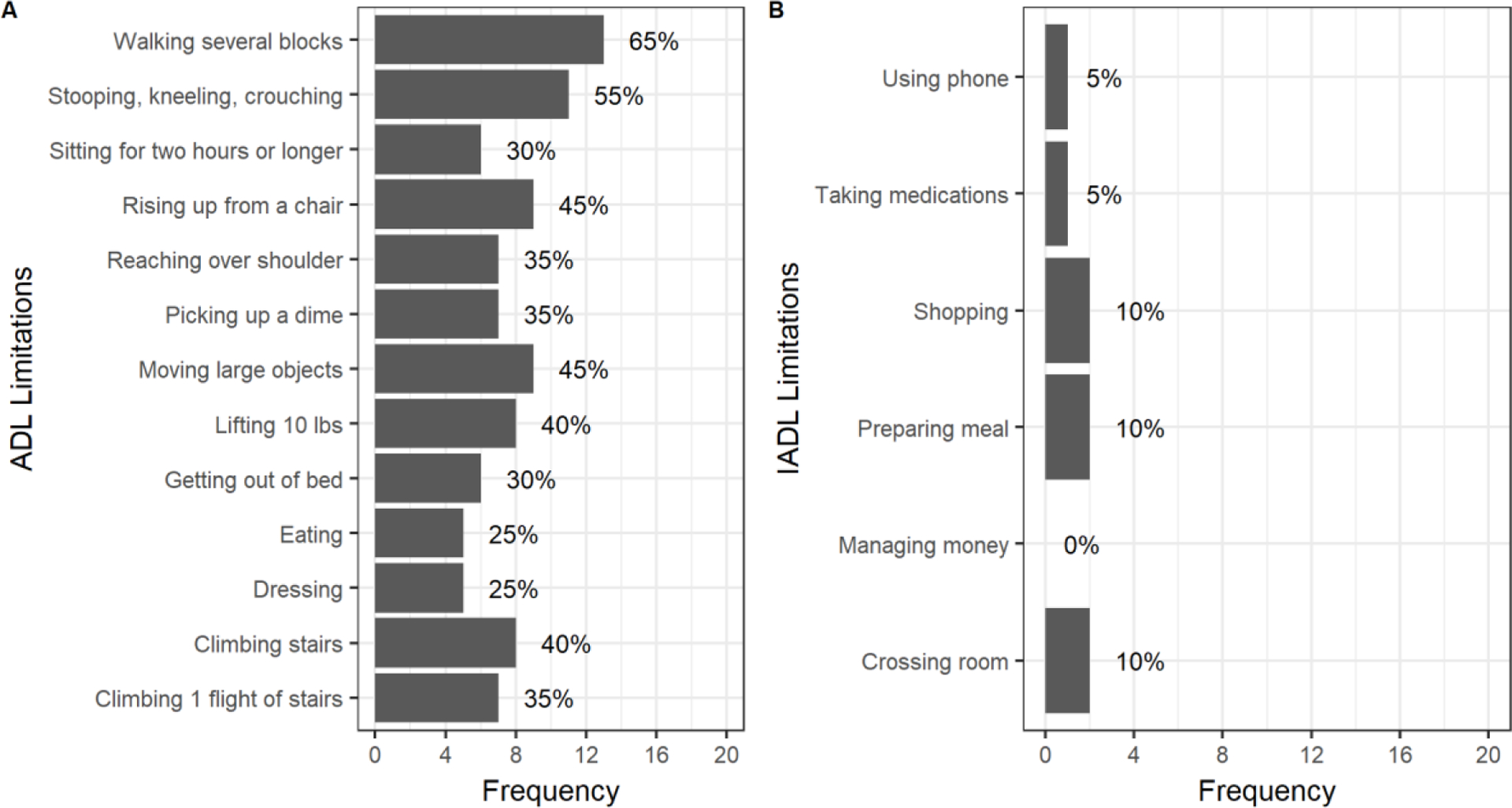
Activities of daily living (ADL) and instrumental activities of daily living (IADL) in older women with urinary incontinence

Self-reported functional status evaluation revealed most prevalent ADLs reported by women were difficulty in walking several blocks (65%) and stooping, kneeling, crouching (55%) (Figure 2). Functional limitations for IADL items were not frequently reported.

Spearman correlation coefficients generally showed an inverse association between patient-reported UI and physical performance measures, where higher UI severity and impact on QOL corresponded to lower mobility and function (Table 3). Lower MPPT score (r=−0.46; P=0.04) and worse TUG test performance (r=0.50; P=0.03) was significantly associated with greater impact of UI on QOL as measured by the IIQ-7. The associations between other physical performance and UI measures, including UI episodes on bladder diary, were lower in magnitude, ranged from approximately 0.10–0.40, and were not statistically significant (P>0.05).

## Discussion

In this pilot study of community-dwelling older women with UI, a standardized physical performance measures evaluation demonstrated that 65% and 35% of women had mobility and functional impairments according to the MPPT and SPPB, respectively. Self-reported upper, lower body mobility, and strength impairments are also prevalent. Of the many physical performance measures, lower MPPT score and worse TUG performance were significantly associated with a higher UI impact on QOL based on IIQ-7.

A recent literature review identified that common geriatric syndromes consistently shared four risk factors: age ≥ 65 years, impaired mobility, functional and cognitive impairments [5]. While it is extremely difficult to study the pathophysiology of complex multifactorial geriatric syndromes, including UI, the main contribution of this study is that it characterized two of these shared risk factors in older women with UI utilizing objective measures. Our protocol consisted of multiple physical performance measures to evaluate mobility and functional impairments. Since the definition of a geriatric syndrome implies a multifactorial condition with impairments in multiple systems, comprehensive description of older women with UI with respect to mobility and functional impairments is necessary in order to understand and define UI geriatric syndrome in older women. The significant negative associations between MPPT, TUG and UI severity suggest these physical performance measures as potential markers of mobility and functional impairments in older women with UI. We are currently conducting a larger study to confirm these findings in search to better understand the differences between UI geriatric syndrome and pelvic floor disorder in older women.

Findings from this pilot clinical study add to emerging evidence from large epidemiologic studies that demonstrated a relationship between UI symptoms and functional impairments in older women. Goode and colleagues assessed predictors of incident UI over 3 years in community-dwelling older women, and found that slower time on 5 chair stands was significantly associated with UI onset [30]. Furthermore, prior studies have shown that gait speed and balance are correlated with the presence of UI [9–12]. Albeit similar, results from the current study did not yield statistically significant associations between gait speed, balance, chair rise and UI. This incongruency may be related to better physical performance (i.e., healthier) characteristics of the current study sample. Alternatively, the small number of participants (n=20) may have resulted in increased measurement variability and inadequate statistical power to assess correlations between certain physical performance and UI variables. Nonetheless, gait speed was strongly correlated with SPPB, MPPT, and TUG (r >0.75; P<0.01) in our sample. Two of these measures were significantly associated with UI impact on QOL based on IIQ-7, and thus, motivate further research.

MPPT and SPPB are composite measures of physical performance. The SPPB measures mobility and predicts mortality, nursing home admissions, and health care utilization among older adults [16], but exhibits ceiling effects and may not detect early functional decline. By contrast, the MPPT is more determinative of early functional decline in aging populations [23]. The current pilot results are consistent with Addison et al. study that suggested that the MPPT may be the more appropriate tool to assess early mobility and functional impairments. Our current research strategy is to focus on further identifying mild mobility and functional impairments in older women with UI and to evaluate a multimodal exercise program as a novel clinical approach to prevent worsening of mobility and functional impairments, and therefore, also decrease symptoms of UI. Older women with greater UI impact on QOL and worse performance based on MPPT and TUG could represent the phenotypic subtype of older women with UI to most benefit from UI treatment paradigm shift to a multidisciplinary treatment approach to improve impairments in multiple systems and treat UI geriatric syndrome. While MPPT consists of nine physical performance measures that could be employed in research, TUG is feasible in a busy urogynecologic clinic to screen older women with UI for mobility and functional impairments. TUG takes 10.4±7.3 seconds based on our study results, it only requires an armchair, stopwatch, and a hall to walk 3 meters. This test could be performed in the clinic while taking other vital signs.

There are several limitations in this pilot study, including the small sample size, lack of comparison group comprising older women without UI, and cross-sectional design. We did not evaluate polypharmacy that can affect both UI and function. However, the participants had low prevalence of chronic illnesses. Most importantly, the pilot aimed to perform comprehensive standardized physical performance measures evaluation among older women with UI. The data from this pilot provides necessary data for larger studies and sample calculations when physical performance measures are evaluated in community-dwelling older women with UI. More broadly, this pilot intended to highlight and facilitate the identification and recognition of mobility and functional impairments in this population and add a different dimension to clinical UI assessment. Other strengths include utilizing a multi-disciplinary research protocol (urogynecology, geriatrics, gerontology, and exercise physiologist) with rigorous screening and strict selection criteria to exclude mobility and functional impairments due to uncontrolled chronic medical, neurologic, and musculoskeletal conditions. Our participants were racially diverse. Majority of women were recruited from Baltimore communities and only 20% women were patients from urogynecologic clinics. Collectively, these attributes enhance the internal validity and generalizability of this pilot study’s findings, and our results represent a reasonable estimate of mobility and functional impairments among older women with UI assessed via physical performance measures.

While treating UI is the overarching goal in urogynecologic care, our pilot underscores high prevalence of mobility and functional impairments in older women with UI. This calls for clinicians to consider broadening current medical management of UI to decrease mobility and functional impairments and lessen UI impact on QOL in this population. Older women with UI, decreased physical performance, and greater UI impact on QOL may constitute a unique clinical phenotype of older women with UI requiring a multidisciplinary intervention approach to improve impairments in multiple systems resulting in decreased UI severity and improved QOL.

## Figures and Tables

**Table 1.** Demographics and Clinical Characteristics*

Variable	Mean	SD
Age, years	76.6	4.7
Body Mass Index (kg/m^2^)	33.5	9
Mini-Mental Status Examination Score	28.3	1.2
	N	%
White	10	50
Hispanic	2	10
Vaginal Deliveries	15	75
Cesareans	2	10
Nulliparity	3	15
Hormonal Therapy	3	15
Prolapse Surgery	0	0
Anticholinergic Incontinence Treatment	7	35
Incontinence Surgery	1	5
Incontinence Symptoms > 1 years	17	85
Incontinence Symptoms > 5 years	9	45
Sexually Active	4	20
Hysterectomy	11	55
Hypertension	14	70
Arthritis	15	75
Hip Fracture	1	5
Diabetes	4	20
Cancer	6	30
Hearing Impairment	1	5

* Study included total of 20 women

**Table 2.** Urinary Incontinence Symptom Severity and Impact on Quality of Life, Urogynecologic Evaluation, and Physical Performance Measures

3-Day Bladder Diary		
UI Episodes, mean (SD)	15.9	9.9
≥ 2 UI Episodes Daily, mean (SD)	18	90
Urgency UI, n (%)	3	15
Stress UI, n (%)	0	0
Mixed UI, n (%)	17	85
Urogynecologic Examination	N	%
Prolapse (above hymen)	4	20
Vaginal Atrophy	19	95
Urethral Hypermobility	1	5
UI Severity & Symptoms	Mean (SD)	Median (IQR)
PGI-S	2.8 (0.6)	3.0 (2 – 4)
Stress UI (MESA)	49.5 (30.3)	57.5 (0–100)
Urgency UI (MESA)	42.3 (22.7)	36.0 (6–78)
UDI-6	42.8 (17.9)	44.4 (11.1–72.2)
IIQ-7	37.3 (27.1)	40.4 (0–80.9)
Physical Performance	Mean (SD)	Median (IQR)
SPPB (0–12)	9.4 (2.7)	10.00 (3–12)
MPPT (0–36)	26.6 (7.1)	28.00 (7–34)
Chair Rise (seconds)	12.3 (2.9)	11.4 (8.1–19.5)
Book Lift (seconds)	2.4 (1.0)	2.4 (0.6–4.8)
Put On/Remove (seconds)	11.5 (5.0)	10.1 (6.3–25.0)
Pick Up Penny (seconds)	4.1 (2.7)	2.8 (1.3–11.2)
50 Foot Walk (seconds)	18.6 (6.7)	16.7 (10.9–40.3)
Climb One Flight of Stairs (seconds)	7.0 (5.7)	5.3 (3.4–28.5)
6 Minute Walk (feet)	1161 (334)	1195 (400–1675)
Gait Speed (meters/second)	0.9 (0.2)	0.9 (0.4–1.4)
Descend Flight of Stairs (seconds)	7.4 (6.9)	5.59 (3.6–34.1)
FSST (seconds)	13.9 (6.7)	12.0 (7.1–37.0)
Grip Strength (kilograms)	18.9 (6.2)	19.0 (3–31)
TUG (seconds)	10.4 (7.3)	7.7 (4.9–37.9)

UI: Urinary Incontinence

GSI – Patient Global Impression of Severity

MESA: Medical Epidemiologic Social Aspect of Aging

UDI-6: Urogenital Distress Inventory Short Form

IIQ-7: Incontinence Impact Questionnaire Short Form

SPPB: Short Physical Performance Battery

MPPT: Modified Physical Performance Test

FSST: Four Square Step Test

TUG: Timed Up and Go

**Table 3.** Associations between Urinary Incontinence Symptom Severity, Impact on Quality of Life and Physical Performance Measures in Older Women

Variable	UDI-6	IIQ-7	Stress UI MESA	Urgency UI MESA	UI *	PGI-S
SPPB	−0.27	−0.39	−0.33	−0.21	0.01	−0.14
MPPT	−0.33	−**0.46**†	−0.34	−0.22	−0.07	−0.19
TUG	0.26	**0.50**‡	0.33	0.33	−0.09	0.37
Gait Speed	−0.15	−0.38	−0.21	−0.21	−0.18	−0.17
Chair Rise	−0.12	−0.04	0.15	0.06	0.07	−0.21
Hand Grip	−0.15	−0.32	−0.28	−0.34	−0.07	−0.28

UDI-6: Urogenital Distress Inventory Short Form

IIQ-7: Incontinence Impact Questionnaire Short Form

UI: Urinary Incontinence; MESA: Medical Epidemiologic Social Aspect of Aging

PGS-I: Patient Global Impression of Severity

SPPB: Short Physical Performance Battery

MPPT: Modified Physical Performance Test

TUG: Time Up and Go.

* UI episodes based on bladder diary.

† P = 0.04.

‡ P = 0.03.

## APPENDIX A. Physical Performance Measures Protocol

### Modified Physical Performance Test

Participants are given up to two chances to complete each item. Assistive devices are permitted for tasks 6 – 9.

#### 1. Standing Static Balance

- **Feet together**: “Stand still with your feet together as demonstrated for 10 seconds.”
- **Semi Tandem**: “Stand with the heel of one foot placed to the side of the 1^st^ toe of the opposite foot for 10 seconds.” Subject chooses which foot goes forward.
- **Tandem**: “Stand with the heel of one foot directly in front of the other foot, for 10 seconds.” Subject chooses which foot goes forward.

#### 2. Chair Rise:

Use a straight back chair with a solid seat that is 16” high. Ask a participant to sit on the chair with arms folded across their chest. “Stand up and sit down as quickly as possible 5 times, keeping your arms folded across your chest.” Stop timing when the participant stands the 5^th^ time.

#### 3. 50-foot walk test:

Bring a participant to start on a 50-foot walk test course (25 feet out and 25 feet back) and ask a participant, on the command “go” to walk as quickly as she can to the 25-foot mark and back. Time from the command “go” until the starting line is crossed on the way back.

#### 4. Book Lift:

Place a heavy book (approximately 5lbs) on a table in front of a participant. Ask a participant, when given the command “go” to place the book, as quickly as she can, on a shelf above shoulder level. Time from the command “go” until when the book is resting on the shelf. Starting position is with participant’s hands at her side. If a participant has a jacket or cardigan sweater, ask her to remove it. If not, give a participant a lab coat. Ask a participant, on the command “go” to quickly put the coat on completely such that it is straight on her shoulders and then remove the garment completely. Time from the command “go” until the garment has been completely removed. Hint: it is more accurate to time putting on the garment, then pause (pause the stopwatch), then time taking off the garment.

#### 6. Pick up a penny from floor:

Place a penny approximately 12 inches from a participant’s foot on the dominant side. Ask a participant, on the command “go” to pick up the penny from the floor and stand up. This is to be done as quickly as she can; yet allowing for safety and comfort. Time from the command “go” until a participant is standing erect with a penny in hand. If dexterity is a problem, a pen or similar lightweight object can be used

#### 7. Turn 360 degrees:

Ask a participant to turn 360 degrees “as quickly as you can, as you feel comfortable and safe”. Evaluate using the scale on scoring sheet.

#### 8 & 9. Stairs:

Bring a participant to foot of stairs (nine to 12 steps) and ask a participant, on the command ”go” to begin climbing up to a total of 4 flights stairs (as quickly as she can, as she feels comfortable and safe) or until a participant feels tired and wishes to stop. Before beginning this task, alert a participant to a possibility of developing chest pain or shortness of breath and inform a participant to tell you if any of these symptoms occur. You will walk with a participant. Time from the command “go” until a participant’s first foot reaches the top of the first flight of stairs. Go on to record the number of flights (maximum is four) completed (up and down is one flight). Provide a chair for resting when completed. Participants are to complete 4 trials. The first three attempts are timed (on scoring sheet). The final 4th trial is untimed and is only to test if a participant can complete 4 flights. On the scoring sheet, “Climb one flight of stairs”, take the fastest ascent time out of the 3 timed trials. The last item on the scoring sheet is for number of stairs climbed. Participants get 1 point for each flight completed for a maximum of 4 points (=4 flights of stairs).

### Modified Physical Performance Test Scoring

**Table:**

1.	Standing Static Balance	Feet Together: sec.	Semi Tandem: sec.	Tandem: sec.		Score
		10s.	10s.	10s.		4
		10s.	10s.	3–9.9s.		3
		10s.	10s.	0–2.9s.		2
		10s.	0–9s.	Unable		1
		0–9s.	Unable	Unable		0

		Time		Scoring values		Score
2.	Chair rise			≤ 11 sec 11.1--14 sec 14.1--17 sec >17 sec unable	= 4 = 3 = 2 = 1 = 0	
3.	Lift a book and put it on a shelf			≤ 2 sec 2.1--4 sec 4.1-- 6 sec > 6 sec unable	= 4 = 3 = 2 = 1 = 0	
4.	Put on and remove a jacket			≤ 10 sec 10.1 --15 sec 15.1 – 20 sec >20 sec unable	= 4 = 3 = 2 = 1 = 0	
5.	Pick up a penny from floor.			≤ 2 sec 2.1--4 sec 4.1-- 6 sec > 6 sec unable	= 4 = 3 = 2 = 1 = 0	
6.	Turn 360 degrees	Discontinuous steps Continuous steps			= 0 = 2	
		Unsteady (grabs, staggers) Steady		= 0 = 2	
7.	50-foot walk test.			≤ 15 sec 15.1--20 sec 20.1--25 sec >25 sec unable	= 4 = 3 = 2 = 1 = 0	
8.	Climb one flight of stairs.			≤ 5 sec 5.1--10 sec 10.1 – 15 sec >15 sec unable	= 4 = 3 = 2 = 1 = 0	
9.	Climb stairs.	**Number of flights of stairs up and down (maximum 4)**				
**TOTAL SCORE**		**9-item score**				**/36**

### Short Physical Performance Battery

#### 1 & 2. Standing Static Balance and Chair Rise

Standing Static Balance and Chair Rise was performed and scored once for both MPPT and SPPB.

#### 3. Gait Speed:

50-foot walk test results were converted to gait speed. 50 feet = 15.24 meters. Gait Speed = 15.24 meters/Time needed to complete 50-foot walk (seconds). Gait speed scores were assigned according to the following table:

**Table:**

Gait Speed (meters/second)	Points
>0.83	4 points
0.83–0.65	3 points
0.64–0.46	2 points
<0.46	1 point
Unable to do	0 points

### Six Minute Walk Test

1. Equipment: Stopwatch, 2 traffic cones, arm chair, clipboard
2. Place cones in the center of the hallway 100 feet apart
3. Participant instructions: “This is the 6-minute walk test. The objective of the test is to walk as far as possible in 6 minutes. This is a self-paced test but the further you walk the better. The cones are 100 feet apart. You will line up next to this first cone and when I say “go” you will walk to the other cone, around it, come back, and walk around this cone and you are just doing laps. As many as you can in 6 minutes. Now, 6 minutes is a long time to walk. If you need to stop during the test you are allowed to stop, rest, sit down, lean against the wall. All of that is permissible but the timer will continue to run so please start walking again if you can as soon as possible. I will keep track of the number of laps that you complete so you just focus on walking as far as you can. I will let you know as each minute goes past, and then at 6 minutes I will ask you to stop where you are.”
4. At “go” start the timer and track the number of laps a participant completes. One lap is when a participant reaches the cone. Monitor a participant for any signs or symptoms of distress. Use the following standard encouragements during the test:
  - 1 min “You are doing well. You have 5 minutes to go.”
  - 2 min “Keep up the good work. You have 4 minutes to go.”
  - 3 min “You are doing well. You are halfway.”
  - 4 min “Keep up the good work. You have only 2 minutes left.”
  - 5 min “You are doing well. You have only 1 minute to go.”
  - 6 min “Please stop where you are.”
5. At the end of the 6-minute walk test put a marker on the distance for any uncompleted lap. Have a participant sit and rest while you measure the excess distance and tally up the total distance.

* Normally the clinician will not walk with the subject to avoid any problems with setting the pace however if a participant is a fall risk the clinician may walk behind a participant.

### Four Square Step Test

Participant instructions: “This next test will test your balance and your ability to change directions. Try to complete the sequence as fast as possible without touching the sticks. Both feet must make contact with the floor in each square. If possible, face forward for the entire sequence.”

The stepping sequence is (clockwise): square 1, square 2, square 3, square 4. Then (counterclockwise): back to square 3, square 2, and end in square 1. One practice trial is allowed. Two trials of the best are performed.

TRIAL 1: _____

TRIAL 2: _____

AVG. SCORE: _____

### Timed Up and Go (TUG) Test

1. Equipment: arm chair, traffic cone, stop watch.
2. Begin the test with a participant sitting correctly (hips all of the way to the back of the seat) in a chair with arm rests. The chair should be stable and positioned such that it will not move when a participant moves from sit to stand. A participant is allowed to use the arm rests during the sit to stand and stand to sit movements.
3. Place a piece of tape or other marker on the floor 3 meters away from the chair so that it is easily seen by a participant.
4. Instructions: “On the word GO you will stand up, walk to the line on the floor, turn around and walk back to the chair and sit down. Walk at your regular pace.”
5. Start timing on the word “GO” and stop timing when a participant is seated again correctly in the chair with her back resting on the back of the chair.
6. A participant wears her regular footwear, may use any gait aid that she normally uses during ambulation, but may not be assisted by another person. There is no time limit. They may stop and rest (but not sit down) if a participant needs to.
7. Normal healthy elderly usually complete the task in ten seconds or less. Very frail or weak elderly with poor mobility may take 2 minutes or more.
8. A participant should be given a practice trial that is not timed before testing.
9. Results correlate with gait speed, balance, functional level, the ability to go out, and can follow change over time.

### Grip Strength Evaluation

1. Equipment: Chair, grip strength isokinetic dynamometer.
2. Begin the test with a participant sitting correctly (upright posture, feet hip width apart on floor, forearms resting on armrest).
3. Hand grip strength isokinetic dynamometer to a participant in one hand.
4. Instructions: “Hold device in one hand. On the word ‘GO’, squeeze the handle as hard as you can for a total of 5 seconds. Don’t stop until the word ‘STOP’”.
5. Each hand will be tested 4 times. Complete 4 tests on one hand prior to switching to next hand.
6. A participant should be allowed the same rest time between each test (10 seconds).
7. Tester will record maximum muscle contraction (peak torque) that is populated by grip strength isokinetic dynamometer after each test.
8. A participant should be given a practice trial that is not timed before testing.
9. Results measure grip strength that correlate with functional level and can follow changes over time.
10. Best performance out of all attempts was included in analysis.
